# Enhancing patient-centric care: the role of PROMs utilizing SRS-30 in pediatric scoliosis management

**DOI:** 10.1186/s41687-025-00904-2

**Published:** 2025-07-01

**Authors:** Marina Rosa Filezio, Ramyn Jooma, Paul Fairie, David Parsons, Maria J. Santana

**Affiliations:** 1 Department of Community Health Sciences, University of Calgary, Calgary, AB Canada; 2 Department of Surgery, University of Calgary, Calgary, AB Canada; 3 School of Kinesiology, University of British Columbia, Vancouver, BC Canada; 4 Department of Pediatrics, University of Calgary, Calgary, AB Canada

**Keywords:** Scoliosis, Pediatric, Surgery, SRS-30, PROMs

## Abstract

**Background:**

Scoliosis is defined by a curvature of the spine greater than 10 degrees. The most common type of scoliosis is called Adolescent Idiopathic Scoliosis and is found in individuals between 11 to 18 years of age. It corresponds to 90% of the cases of scoliosis in the pediatric population, with an overall prevalence of 0.47–5.2%, affecting girls more than boys (3:1). There are different treatment options for scoliosis, and surgery is reserved for patients with curves greater than 45 degrees while still growing or greater than 50 degrees for skeletally mature patients. There is a growing recognition of the important role of patient-reported outcomes measures (PROMs) for understanding the impact of scoliosis on individuals’ lives and its management. This paper explores the importance of PROMs, specifically the Scoliosis Research Society-30 (SRS-30) questionnaire, in assessing and improving the quality of care for pediatric scoliosis patients that were submitted to surgical intervention.

**Methodology:**

PROMs data were collected at predefined time points: pre-operatively (baseline), and post-operatively at 3, 6, and 12 months. The evaluation encompassed 23 (pre-operative assessment) to 30 questions (follow-up) and included five key domains: Function/Activity, Pain, Self-Image/Appearance, Mental Health, and Satisfaction with Management, as well as possible changes in the results before and after surgery.

**Results:**

115 patients participated in this study, of whom 79% were females (mean age 14.5 years). Function/Activity was the only domain to exhibit a significant score decrease in the post-operative follow-up, with a return to baseline levels at the 12-months mark. All other domains showed statistically significant improvement over time, with the steepest increase observed at 3 months for Self-Image/Appearance and Satisfaction with Management. Age did not significantly influence on the results across any of the five domains.

**Conclusions:**

This project highlights the pivotal role of PROMs, with a specific focus on the SRS-30 questionnaire results, in creating a more holistic and patient-centered approach to scoliosis management.

**Supplementary Information:**

The online version contains supplementary material available at 10.1186/s41687-025-00904-2.

## Background

Scoliosis is the most common spinal disorder seen in pediatric patients and is the medical term that defines a curvature of the spine greater than 10 degrees. The most common type of scoliosis is called Adolescent Idiopathic Scoliosis (AIS) and is found in individuals between 11 to 18 years of age. AIS corresponds to 90% of the cases of scoliosis in the pediatric population, with an overall prevalence of 0.47–5.2%, affecting girls more than boys (3:1) [1, 2].

There are different treatment options for scoliosis, depending mainly on the curvature size and skeletal maturity [3, 4]. Surgery is reserved for patients with curves greater than 45 degrees while still growing or greater than 50 degrees for patients that have completed their growth [5]. There are several surgical techniques that could be used for scoliosis correction, but the most performed worldwide is the Posterior Spinal Instrumentation and Fusion (PSIF), where the spine is assessed by a posterior approach and the curvature is corrected and fixed with the help of surgical hooks, screws, and rods [6–8]. Surgical treatment can control curve progression, improve pain, trunk appearance, and self-image [9, 10].

Traditionally, surgical success is often based on the physicians’ post-operative assessment, including curvature correction and adequate instrumentation placement [11]. As healthcare continues to evolve, there is a growing recognition of the need to prioritize patient-reported outcomes (PROMs) for a comprehensive understanding of the impact of surgical procedures on individuals’ lives [12]. A majority of PROMs available in the literature were developed for adult respondents, with a smaller number of validated pediatric questionnaires [13, 14]. The Scoliosis Research Society (SRS) created a range of Health-Related Quality of Life Tools, including validated questionnaires for the pediatric population such as the SRS-30, which consists of 30 questions that measure scoliosis-specific health-related domains [9–13].

By utilizing validated, scoliosis-specific PROM, this study explores how PSIF for AIS can impact patients’ quality of life, providing a comprehensive understanding of surgical outcomes beyond the traditional clinical metrics. The aims of this study are to:

1. Assess baseline PROM pre-operatively.
2. Investigate changes in PROM post-surgery at multiple time points (3 months, 6 months and 1 year).

## Methods

This was a single center, prospective cohort study, conducted at the Alberta Children’s Hospital Pediatric Spine clinic (Calgary, AB, Canada) between August 2020 and February 2024. Patients with AIS, between the ages 11 to 18, that were schedule to undergo PSIF, were eligible to participate in the study. Patients who did not speak/read English (or whose parents did not speak/read English), or with non-idiopathic scoliosis, or who were schedule for an AIS PSIF revision surgery were excluded from enrolling in this study. Participants were not excluded for missing data, such as not completing the PROM in all four time points.

This study received ethics approval from the University of Calgary Research Ethics Board (REB#18-0047)

Patients were identified and offered to complete the PROM (Appendix A) at four different time points (pre-operatively, 3-, 6- and 12-months post-operatively). PROM was provided either in paper format during in-hospital assessment or via email, depending on their preference and clinical protocol at the time. The pre-operative aspect of the PROM encompasses 23 multiple-choice questions, while the post-operative includes 7 extra post-operative specific multiple-choice questions (30 in total). Each question scored on a scale of 1 (worst) to 5 (best). The PROM comprises five key domains: Function/Activity, Pain, Self-Image/Appearance, Mental Health, and Satisfaction with Management. The number of questions for each domain is represented in Table 1.

**Table 1:** Number of specific questions per domain pre-operatively and post-operatively, in the SRS-30 questionnaire

Domain	Pre-operative questions (N)	Post-operative specific questions (N)	Total questions per domain (N)
Function/Activity	5	2	7
Pain	5	1	6
Self-Image/ Appearance	6	3	9
Mental Health	5	0	5
Satisfaction with Management	2	1	3

Each distinct domain captures key dimensions of patients’ experiences. The **Function/Activity** domain evaluates the patient’s ability to perform physical tasks and participate in age-appropriate activities, including school and social life**. Pain** assesses the frequency and intensity of back pain and its impact on daily activities. **Self-Image/Appearance** reflects the patient’s perception of their physical appearance and body image in the context of scoliosis. The **Mental Health** domain captures emotional well-being, including mood, anxiety, and self-esteem. Finally, **Satisfaction with Management** measures the patient’s satisfaction with their treatment and its perceived outcomes.

The scoring for each PROM was done following the Score Sheet instructions provided by the SRS (Appendix B).

Adjustments were made for both age and sex to determine whether specific outcomes were more common for certain age groups or sex. The goal of controlling for these variables was to identify any age- or sex-related patterns or variations in the results. This adjustment allows for a more precise understanding of how age and sex may influence PROMs for AIS patients undergoing PSIF, ensuring that the analysis accounts for potential differences and provides more specific insights regarding post-operative expectations and outcomes.

*Primary Outcomes:* The pre-operative score was deemed as the patient’s PROM baseline, providing information of how AIS can influence patients’ quality of life prior to treatment. Moreover, the post-operative score was used to observe possible changes in PROM post-operatively, looking into the direct comparison between pre- and post-operative scores, as well as possible changes over time.

*Secondary Outcomes:* The data obtained help tailor AIS management according to patients’ specific characteristics, as well as better inform patients and families about the post-operative expectations surrounding surgical intervention.

A linear mixed model (LMM) analysis was conducted on each domain category. LMM was chosen for statistical analysis due to their ability to handle repeated measures data with missing values, account for both fixed effects (e.g. age and sex) and random effects in PROM scores over multiple time points while retaining statistical power despite unequal follow-up completion rates. Statistical analysis was performed using statistical software R (version 4.2.2).

## Results

A total of 125 potential participants consented to take part in this study. Of these, 115 participants completed at least one PROM and were included in the analysis. The majority of participants were female (78%), with a mean age of 14.6 years (SD = 1.7 and range of ages = 7 years). The distribution of questions across the five key domains of the PROM is shown in Table 1. Basic demographic data (age and biological sex) were collected to explore possible trends according to participants’ characteristics (Table 2).

**Table 2:** Demographic data from the study participants N (%)

Sex	
Female	91 (79,1%)
Male	24 (20.9%)
Age	
11 years old	5 (4.3%)
12 years old	8 (6.9%)
13 years old	13 (11.3%)
14 years old	31 (26.9%)
15 years old	22 (19.1%)
16 years old	17 (14.8%)
17 years old	17 (14.8%)
18 years old	2 (1.7%)

The questionnaire completion was as follows: pre-operative baseline, 78% (90); 3 months follow-up, 23% (27); 6 months follow-up 36% (42); and 1 year follow-up 37% (43). A total of 202 PROM results were included in this study.

In order to better visualize the results, they will be presented according to each domain.

### Function/Activity

**Fig. 1:**
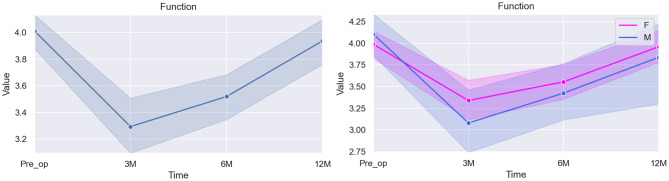
Graphical illustration of the overall score for Function (left) and the specific differences between scores according to sex (right)

The statistical results for the domain “Function/Activity” are summarized in Table 3 and Fig. 1:

**Table 3:** Mixed linear model regression results for function/activity

Variable	Coef.	Std. Err.	p-value	CI [97.5%]
Pre-op (baseline)	4.722	0.395	0.000	3.949–5.496
3 months post-op	−0.793	0.116	0.000	−1.021–0.566
6 months post-op	−0.520	0.098	0.000	−0.712–0.328
12 months post-op	−0.126	0.098	0.201	−0.319–0.067
Sex*	0.009	0.107	0.936	−0.201–0.218
Age	−0.045	0.027	0.092	−0.098–0.007

* The baseline used for the category Sex was Female

The statistical analysis presented an intercept coefficient of 4.722, representing the estimated baseline level of patient function at the starting point of our analysis. Statistically significant (*p < 0.005*) decrease in this domain can be seen at 3 months (−0.793, *p < 0.001*) and 6 months (−0.520, *p < 0.001*), with no significant changes at the 12-months mark (−0.126, *p = 0.201*) in comparison to baseline.

The coefficient for Sex (0.009), suggests minimal and not statistically significant (*p = 0.936*) difference in patient function between male and female patients. Similar results can be seen when looking into the variable Age (*p = 0.092*).

### Pain

**Fig. 2:**
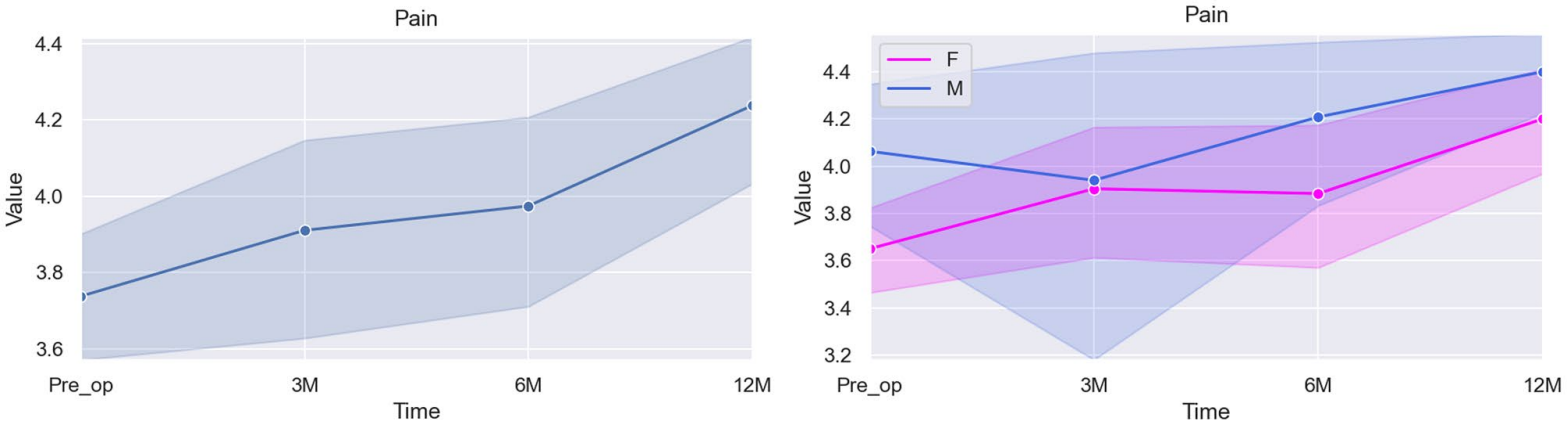
Graphical illustration of the overall score for pain (left) and the specific differences between scores according to sex (right)

Table 4 and Fig. 2 present the statistical results obtained for Pain:

**Table 4:** Mixed linear model regression results for pain

Variable	Coef.	Std. Err.	p-value	CI [97.5%]
Pre-op (baseline)	4.315	0.582	0.000	3.174–5.456
3 months post-op	0.130	0.149	0.383	−0.162–0.422
6 months post-op	0.213	0.125	0.087	−0.031–0.458
12 months post-op	0.453	0.127	0.000	0.203–0.702
Sex*	0.389	0.159	0.014	0.078–0.700
Age	−0.045	0.040	0.254	−0.123–0.033

* The baseline used for the category Sex was Female

The analysis showed an estimated baseline score of 4.315 for this domain pre-operatively. A slight improvement in pain levels was observed at 3 months (0.130), but this change was not statistically significant (*p = 0.383*). At 6 months, the improvement increased (0.213), though it remained non-significant (*p = 0.087*). By 12 months, a substantial and statistically significant improvement was evident (0.453, *p < 0.001*).

A difference was also observed between male and female patients in this domain, with males reporting slightly higher overall pain scores—indicating less pain—compared to females (*p = 0.014*). Figure 2 further illustrates these differences by sex: male patients reported better pain scores both at baseline and at the 12-month follow-up.

Age, once again, demonstrated no influence in the results for this domain (*p = 0.254*).

### Self-image/appearance

**Fig. 3:**
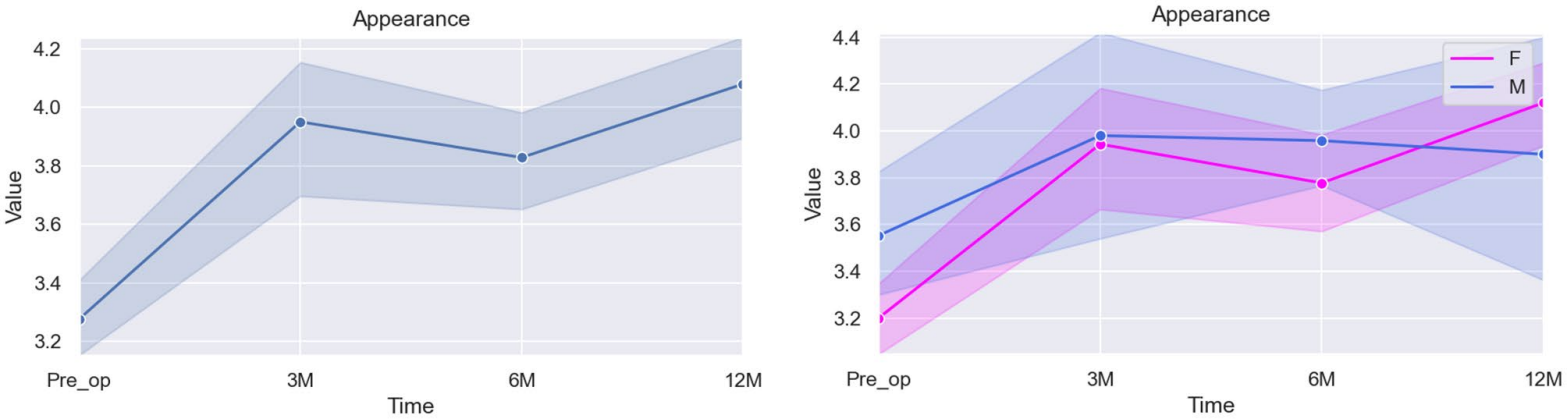
Graphical illustration of the overall score for Self-Image/Appearance (left) and the specific differences between scores according to sex (right)

The statistical analysis results for this domain are illustrated in Table 5 and Fig. 3, as follows:

**Table 5:** Mixed linear model regression results for self-image/appearance

Variable	Coef.	Std. Err.	p-value	CI [97.5%]
Pre-op (baseline)	3.507	0.483	0.000	2.561–4.454
3 months post-op	0.596	0.114	0.000	0.372–0.819
6 months post-op	0.514	0.095	0.000	0.328–0.700
12 months post-op	0.736	0.098	0.000	0.545–0.927
Sex*	0.186	0.133	0.162	−0.075–0.447
Age	−0.018	0.033	0.586	−0.083–0.047

* The baseline used for the category Sex was Female

The estimated baseline score for Self-Image/Appearance in the pre-operative time is 3.507. This domain presented statistically significant improvement (*p < 0.001*) at all post-operative times (0.596 at 3 months, 0.514 at 6 months, and 0.736 at 12 months).

No statistically significant difference in the results was seen when looking into the variables sex and age (*p = 0.162* and *p = 0.586*, respectively).

### Mental Health

**Fig. 4:**
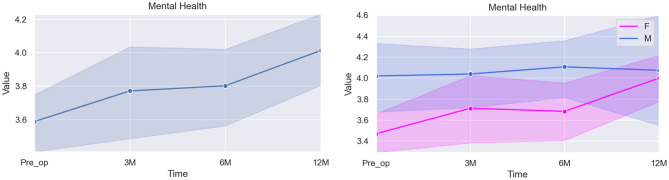
Graphical illustration of the overall score for Mental Health (left) and the specific differences between scores according to sex (right)

Table 6 and Fig. 4 present above the statistical results for this domain:

**Table 6:** Mixed linear model regression results for mental health

Variable	Coef.	Std. Err.	p-value	CI [97.5%]
Pre-op (baseline)	4.620	0.604	0.000	3.436–5.804
3 months post-op	0.130	0.115	0.259	−0.096–0.356
6 months post-op	0.190	0.095	0.047	0.003–0.376
12 months post-op	0.493	0.168	0.001	0.127–0.516
Sex*	0.493	0.168	0.003	0.165–0.822
Age	−0.076	0.041	0.065	−0.158–0.005

* The baseline used for the category Sex was Female

The estimated baseline value for Mental Health was 4.620. At 3 months post-op, a slight increase is seen in the scores (0.130), with no statistical significance seen when compared to baseline (*p = 0.259*). However, at 6 months, the score improvement approaches significance (0.190, *p = 0.047*), with further significant improvement seen at the 12-months mark (0.322, *p = 0.001*).

In this domain, the variable sex is statistically significant (p = 0.003), with males reporting higher Mental Health scores than females. Age does not appear to have significant influence in this domain (*p = 0.065*).

### Satisfaction with Management

**Fig. 5:**
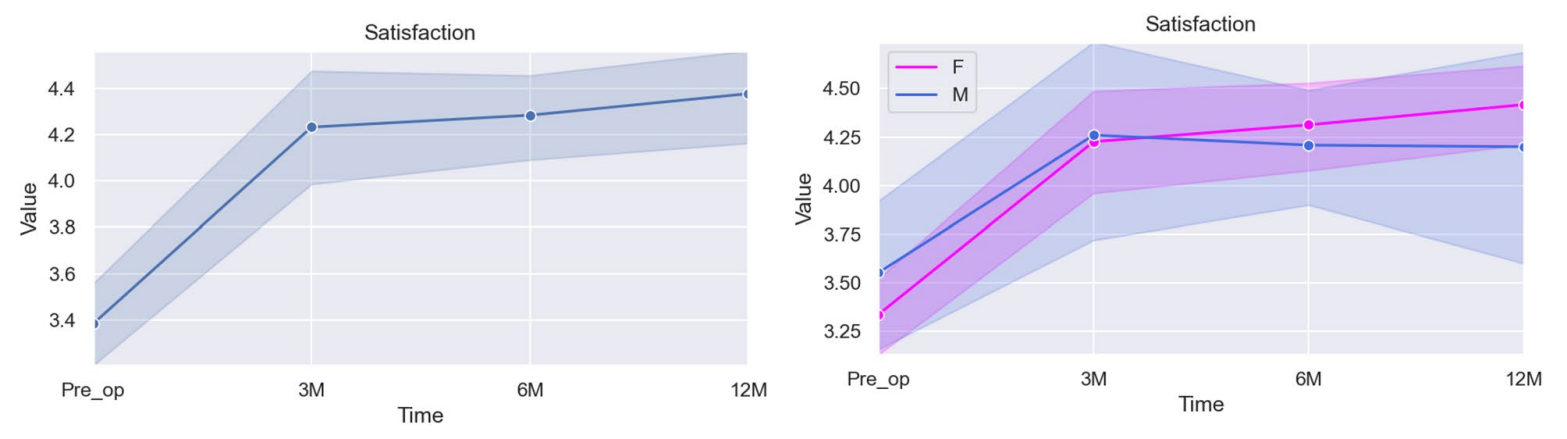
Graphical illustration of the overall score for Satisfaction with Management (left) and the specific differences between scores according to sex (right)

The results for the Satisfaction with Management domain are presented in Table 7 and Fig. 5:

**Table 7:** Mixed linear model regression results for satisfaction with management

Variable	Coef.	Std. Err.	p-value	CI [97.5%]
Pre-op (baseline)	3.694	0.585	0.000	2.547–4.841
3 months post-op	0.831	0.150	0.000	0.538–1.125
6 months post-op	0.907	0.126	0.000	0.661–1.153
12 months post-op	0.957	0.128	0.000	0.706–1.207
Sex*	0.037	0.160	0.818	−0.278–0.351
Age	−0.022	0.040	0.589	−0.100–0.057

* The baseline used for the category Sex was Female

The estimated baseline level for patient’s Satisfaction with Management was set at 3.694. A substantial and statistically significant increase is seen at the 3-months mark (0.831, *p < 0.001*), which continues at 6- and 12-months port-op (0.907 and 0.957, respectively; *p < 0.001*).

No statistically significant difference is seen between males and females (*p = 0.818*) or according to age (*p = 0.589*).

## Discussion

The results presented suggest that patient Function/Activity decreases in the initial post-operative period, with significant decrease observed at 3- and 6-months post-op. However, by the 12-months mark, the score returns to very similar values to the ones presented at baseline. These findings support the post-operative routine for AIS patients submitted to PSIF, where they are recommended to not participate in activities or sports for at least the first 6 months post-op. Moreover, the results found in our study agree with the literature. Pellegrino et al. found decreased function at 3-month follow-up, with significant improvement by the 12-months mark in a similar population (94% female, with a mean age of 15.6 years) [15].

The findings from this study highlight the importance of regularly assessing patient function and quality of life through PROMs such as the SRS-30. Overall, PROMs are invaluable tools in assessing the impact of surgical interventions on patients’ lives and function, contributing to better patient-centered care. Understanding the changes in the five key domains through PROMs allows clinicians to monitor post-operative recovery closely and make outcome predictions and timely interventions to improve patient outcomes.

Investigating the Pain domain, we observed notable trends suggesting improvements in patient-reported pain levels, even in the initial post-operative period, although these changes did not reach statistical significance. However, there was a consistent improvement in pain scores over time following surgical intervention, culminating in a statistically significant improvement by 12 months post-op, aligning with findings reported in the existing literature [9, 15]. Notably, our study revealed a pre-operative difference according to sex, with females reporting significantly worse pain levels than males, consistent with previous research by Ng et al. [16].

Similarly, our examination of the Mental Health domain also demonstrated a trend of improvement over time, with significantly higher scores observed at 12 months post-op in comparison to baseline. Once again, sex emerged as a noteworthy factor, with female patients consistently reporting worst baseline mental health scores than males.

Self-Image/Appearance and Satisfaction with Management scores showed significant improvement over time following the surgical procedure, with substantial increases observed at 3 months port-op and continuous improvement at 6 and 12 months. Ng et al. demonstrated that male patients reported poorer self-image before surgery [16], a correlation that was not observed in our study population, as sex did not significantly influence appearance scores.

The results of this study underscore the importance of PROMs in assessing and improving the quality of care for pediatric scoliosis patients. By tracking the five key domains, clinicians can obtain a comprehensive view of the patient’s recovery trajectory, identify possible signs of complications, and tailor interventions to each individual patient’s need. Moreover, the inclusion of PROMs in clinical practice allows for continuous feedback from patients, fostering shared decision-making and personalized care plans that could contribute to better long-term outcomes.

The findings of this study should be considered in the context of several study limitations. First, there was an important inconsistency of follow-up questionnaire completion, with lower rates observed specially at the 3-month follow-up (23%), which could induce bias and limit the ability to truly assess the effects of the surgical intervention at early follow-up stages. This inconsistency occurred due to changes in administrative personnel during data collection, a transition in the hospital’s medical record system (from paper to electronic charts), and disruptions related to the COVID-19 pandemic (in-person PROMs completed in clinic had to be sent via email due to restrictions on in-person visits). Second, our study focused only on the SRS-30 questionnaire, with no direct correlation to surgical or clinical aspects that might influence the results presented. Third, this was a single center study with a relatively small sample, which might limit the generalizability of our results to different populations.

## Conclusion

The findings of this study offer valuable insights into the trajectory of PROMs for AIS patients undergoing PSIF. These insights have important implications for patient-centered care approaches in AIS management, such as providing patients with more accurate information about post-operative expectations based on sex, a more detailed timeline of post-operative results for each domain, and enhanced resources (including specific physiotherapy assessments and mental health support) to assist patients throughout their post-operative recovery. By examining quality of life domains before and after surgical intervention, this study sheds light on the expected post-operative outcomes for both physicians and patients. Furthermore, the study identifies differences in each domain according to sex, underscoring the importance of considering individual patient characteristics in treatment planning and available support.

Overall, these findings contribute to the existing literature and emphasize the importance of incorporating PROMs into pediatric care and surgical management strategies for AIS.

## Electronic supplementary material

Below is the link to the electronic supplementary material.

Supplementary Material 1

Supplementary Material 2

## Data Availability

The datasets used and analyzed during the current study are available from the corresponding author on reasonable request.
