# Supplementary material for: Enhancing patient-centric care: the role of PROMs utilizing SRS-30 in pediatric scoliosis management
Source: J Patient Rep Outcomes. 2025 Jul 1;9:78. doi: 10.1186/s41687-025-00904-2 (PMC12214136; doi:10.1186/s41687-025-00904-2)
Supplement: Supplementary file 1 — Supplementary Material 1 [file 41687_2025_904_MOESM1_ESM.pdf]

# Scoliosis Patient Questionnaire: Version 30 (Encompasses Versions 22 and 24)

**Modified 06/11/13**

**Patient Name:**________________________ **Age:**______ **Date:**____________

**Medical Record #:** ________________________ **Sex:** ____________

**Exam:** Pre-treatment 3 mos. 6 mos. 1 year ______ years

Your doctors are carefully evaluating the condition of your back before and after your treatment. Please circle the one best answer to each question unless otherwise indicated. If you already have had surgery, please complete **sections 1 and 2.** Otherwise, just complete section **1.**

<u>All results will be kept confidential.</u>

## Section 1: All Patients

**1.** 22:1 24:1 **Which one of the following best describes the amount of pain you have experienced during the past 6 months?**
- ☐ None 5
- ☐ Mild 4
- ☐ Moderate 3
- ☐ Moderate to severe 2
- ☐ Severe 1

**2.** 22:2 24:2 **Which one of the following best describes the amount of pain you have experienced over the last month?**
- ☐ None 5
- ☐ Mild 4
- ☐ Moderate 3
- ☐ Moderate to severe 2
- ☐ Severe 1

**3.** 22:3 24:n/a **During the past 6 months have you been a very nervous person?**
- ☐ None of the time 5
- ☐ A little of the time 4
- ☐ Some of the time 3
- ☐ Most of the time 2
- ☐ All of the time 1

**4.** 22:4 24:3 **If you had to spend the rest of your life with your back shape as it is right now, how would you feel about it?**
- ☐ Very happy 5
- ☐ Somewhat happy 4
- ☐ Neither happy nor unhappy 3
- ☐ Somewhat unhappy 2
- ☐ Very unhappy 1

**5.** 22:5 24:4 **What is your current level of activity?**
- ☐ Bedridden/wheelchair 1
- ☐ Primarily no activity 2
- ☐ Light labor, such as household chores 3
- ☐ Moderate manual labor and moderate sports, such as walking and biking 4
- ☐ Full activities without restriction 5

**6.** 22:6 24:5 **How do you look in clothes?**
- ☐ Very good 5
- ☐ Good 4
- ☐ Fair 3
- ☐ Bad 2
- ☐ Very bad 1

**7.** 22:7 24:n/a **In the past 6 months have you felt so down in the dumps that nothing could cheer you up?**
- ☐ Very often 1
- ☐ Often 2
- ☐ Sometimes 3
- ☐ Rarely 4
- ☐ Never 5

**8.** 22:8 24:6 **Do you experience back pain when at rest?**
- ☐ Very often 1
- ☐ Often 2
- ☐ Sometimes 3
- ☐ Rarely 4
- ☐ Never 5

**9.** 22:9 24:7 **What is your current level of work/school activity?**
- ☐ 100% normal 5
- ☐ 75% normal 4
- ☐ 50% normal 3
- ☐ 25% normal 2
- ☐ 0% normal 1

**10.** 22:10 24:n/a **Which of the following best describes the appearance of your trunk; defined as the human body except for the head and extremities?**
- ☐ Very good 5
- ☐ Good 4
- ☐ Fair 3
- ☐ Poor 2
- ☐ Very poor 1

**11.** 22:11 24:8 **Which one of the following best describes your medication usage for your back?**
- ☐ None 5
- ☐ Non-narcotics weekly or less (e.g., Tylenol, Ibuprofen) 4
- ☐ Non-narcotics daily 3
- ☐ Narcotics weekly or less (e.g., Tylenol #3, Lorocet, Percocet, Darvocet) 2
- ☐ Narcotics daily 1
- ☐ Other *(please specify below)*

Medication: ____________________

Usage (weekly or less or daily): ____________________

12. **Does your back limit your ability to do things around the house?**
22:12
24:9
☐ Never 5
☐ Rarely 4
☐ Sometimes 3
☐ Often 2
☐ Very often 1

13. **Have you felt calm and peaceful during the past 6 months?**
22:13
24:n/a
☐ All of the time 5
☐ Most of the time 4
☐ Some of the time 3
☐ A little of the time 2
☐ None of the time 1

14. **Do you feel that your back condition affects your personal relationships?**
22:14
24:11
☐ None 5
☐ Slightly 4
☐ Mildly 3
☐ Moderately 2
☐ Severely 1

15. **Are you and/or your family experiencing financial difficulties because of your back?**
22:15
24:12
☐ Severely 1
☐ Moderately 2
☐ Mildly 3
☐ Slightly 4
☐ None 5

16. **In the past 6 months have you felt down-hearted and blue?**
22:16
24:n/a
☐ Never 5
☐ Rarely 4
☐ Sometimes 3
☐ Often 2
☐ Very often 1

17. **In the last 3 months have you taken any sick days from work/school due to back pain and, if so, how many?**
22:17
24:10

☐ 0 5 ☐ 1 4 ☐ 2 3 ☐ 3 2 ☐ 4 or more 1

18. **Do you go out more or less than your friends?**
22:18
24:13
☐ Much More 5
☐ More 4
☐ Same 3
☐ Less 2
☐ Much less 1

19. **Do you feel attractive with your current back condition?**
22:19
24:14
☐ Yes, very 5
☐ Yes, somewhat 4
☐ Neither attractive nor unattractive 3
☐ No, not very much 2
☐ No, not at all 1

20. **Have you been a happy person during the past 6 months?**
22:20
24:n/a
☐ None of the time 1
☐ A little of the time 2
☐ Some of the time 3
☐ Most of the time 4
☐ All of the time 5

21. **Are you satisfied with the results of your back management?**
22:n/a
24:15
☐ Very satisfied 5
☐ Satisfied 4
☐ Neither satisfied nor unsatisfied 3
☐ Unsatisfied 2
☐ Very unsatisfied 1

22. **Would you have the same management again if you had the same condition?**
22:21
24:15
☐ Definitely yes 5
☐ Probably yes 4
☐ Not sure 3
☐ Probably not 2
☐ Definitely not 1

23. **On a scale of 1 to 9, with 1 being very low and 9 being extremely high, how would you rate your self-image?**
22:22
24:24

| ☐ 1 ☐ 2 | ☐ 3 ☐ 4 | ☐ 5 | ☐ 6 ☐ 7 | ☐ 8 ☐ 9 |
|---|---|---|---|---|
| 1 | 2 | 3 | 4 | 5 |

## Section 2: Post-surgery patients only

24. **Compared with before treatment, how do you feel you now look?**
22:n/a
24:23
☐ Much Better 5
☐ Better 4
☐ Same 3
☐ Worse 2
☐ Much Worse 1

25. **Has your back treatment changed your function and daily activity?**
22:n/a
24:16
☐ Increased 5 ☐ Not changed 3 ☐ Decreased 1

26. **Has your back treatment changed your ability to enjoy sports/hobbies?**
22:n/a
24:17
☐ Increased 5 ☐ Not changed 3 ☐ Decreased 1

27. **Has your back treatment ____________ your back pain?**
22:n/a
24:18
☐ Increased 1 ☐ Not changed 3 ☐ Decreased 5

28. **Has your treatment changed your confidence in personal relationships with others?**
22:n/a
24:19
☐ Increased 5 ☐ Not changed 3 ☐ Decreased 1

29. **Has your treatment changed the way others view you?**
22:n/a
24:20
☐ Much Better 5
☐ Better 4
☐ Same 3
☐ Worse 2
☐ Much Worse 1

30. **Has your treatment changed your self-image?**
22:n/a
24:21
☐ Increased 5 ☐ Not changed 3 ☐ Decreased 1
