# Supplementary material for: Enhancing patient-centric care: the role of PROMs utilizing SRS-30 in pediatric scoliosis management
Source: J Patient Rep Outcomes. 2025 Jul 1;9:78. doi: 10.1186/s41687-025-00904-2 (PMC12214136; doi:10.1186/s41687-025-00904-2)
Supplement: Supplementary file 2 — Supplementary Material 2 [file 41687_2025_904_MOESM2_ESM.pdf]

SRS-30 Patient Questionnaire/Score Sheet

Name: ______________________ Today's Date: ____________
Mo Day Year

Age: ____ ______
Yr Mo

Sex: M F

Diagnoses: ______________
____
____

Deformity/Size__________ __________

Management: (Circle one)
Initial Evaluation
Observation
Pre Brace
Brace__________
Type
Other __________
Describe

Pre Surgery
Indication__________

	Arthrodesis		Instrumentation	
Surgery	UV	LV	UV	LV
Post	___		___	
Ant	___		___	

Date Initiated: ____ ____ ____
____ ____ ____
____ ____ ____
Mo Day Yr

Follow-up ____ ____
____
____ ____
Yrs Mo

DOMAIN	(Score: 5 Best – 1 Worst)	Post Surgery Questions	Score Pt/Possible(Max) A	#Questions Answered(Possible) B	Mean Score *** A÷B
Function/ Activity	5* 9 12 15 18	25 26	___(__)(25) (35)+	___(5) (7)+	___
Pain	1 2 8 11 17	27	___(__)(25) (30)	___(5) (6)	___
Self Image/ appearance	4 6 10 14 19 23	28 29 30	___(__)(30) (45)	___(6) (9)	___
Mental health**	3 7 13 16 20		___(__)(25)	___(5)	___
SUB TOTAL			___(__)(105) (135)	___(21) (27)	___
Satisfaction with management	21 22	24	___(__)(10) (15)	___(2) (3)	___
TOTAL			___(__)(115) (150)	___(23) (30)	___

+max/possible with post surgery questions

***Mean Score
5 Best
1 Worst

*Question Number
**Questions adopted with permission from SF-36

SCORING INSTRUCTIONS
Unanswered questions – reduce questions answered denominator by appropriate number
Delete questions with more than one response
Domain can't be scored if fewer than 3 questions answered
